# Supplementary material for: Three-Dimensional Paper-Based Microfluidic Analysis Device for Simultaneous Detection of Multiple Biomarkers with a Smartphone
Source: Biosensors (Basel). 2020 Nov 21;10(11):187. doi: 10.3390/bios10110187 (PMC7700338; doi:10.3390/bios10110187)
Supplement: Supplementary file 1 [file biosensors-10-00187-s001.pdf]

Supporting information

Three-dimensional paper-based microfluidic analysis device for simultaneous detection of multiple bio markers with a smartphone

Seung Ho Baek[1,†], Chanyong Park[2, †], Jaehyung Jeon[1] and Sungsu Park[1,2,3,4,*]

[1] School of Mechanical Engineering, Sungkyunkwan University, Suwon, 16419, Korea
[2] Department of Biomedical Engineering, Sungkyunkwan University, Suwon, 16419, Korea
[3] Department of Biomedical Engineering, Sungkyunkwan University, Suwon, 16419, Korea
[4] Biomedical Institute for Convergence at SKKU (BICS), Sungkyunkwan University, Suwon, 16419, Korea
[†] These authors contributed equally to this work and should be considered as co-first author

*Corresponding author: nanopark@skku.edu; Tel.: +82-31-290-7431; Fax: +82-31-290-5889

Table S1. Comparison of gray scale intensities measured by ImageJ (NIH, Bethesda, MD, USA) and a smartphone application. The application was custom-made for the study. The gray intensity of each detection zone was measured using either ImageJ or an application of the smartphone. Biomarkers at different concentrations in serum were prepared using Lyphocheck Assayed Chemistry Control (Bio-Rad, Richmond, CA, USA) [1]. In details, a mixture of serum containing the biomarkers at different concentrations were prepared by carefully mixing human serum Lyphocheks Assayed Chemistry Control level 1 and level 2 containing different concentrations of each biomarker. Then, 5 mL of deionized water was added into the mixture to adjust the concentrations of the biomarkers. After the addition, the mixture was gently shaken without generating bubbles and 300 μL of the mixture were dropped onto the sample reservoir of the 3D-μPADs. Chol.: cholesterol. ALP: alkaline phosphatase. AST: aspartate aminotransferase. UN: urea nitrogen. (a.u.: arbitrary units.)

**Biomarker concentration**	Image J gray intensity (a.u.) (A)	Smartphone application gray intensity (a.u) (B)	Relative error (%) **[|A-B|/A*100]**
High glucose (8 mmole/L)	88.8 ± 1.5	90.3 ± 1.5	1.7
High Chol. (8 mmole/L)	34.1 ± 1.1	35.7 ± 2.1	4.3
Normal Albumin (4 g/dL)	49.4 ± 0.7	50.7 ± 1.5	2.5
High ALP (400 U/L)	30.2 ± 0.1	30.3 ± 2.1	0.4
High Creatinine (150 μmole/L)	34.8 ± 0.6	35.7 ± 0.6	0.5
High AST (400 U/L)	27.5 ± 0.6	28 ± 1.1	1.7
High ALT (400 U/L)	26.3 ± 1.1	26.7 ± 1.5	1.5
Normal UN (4 mmole/L)	55.7 ± 0.6	53.3 ± 0.6	4.6

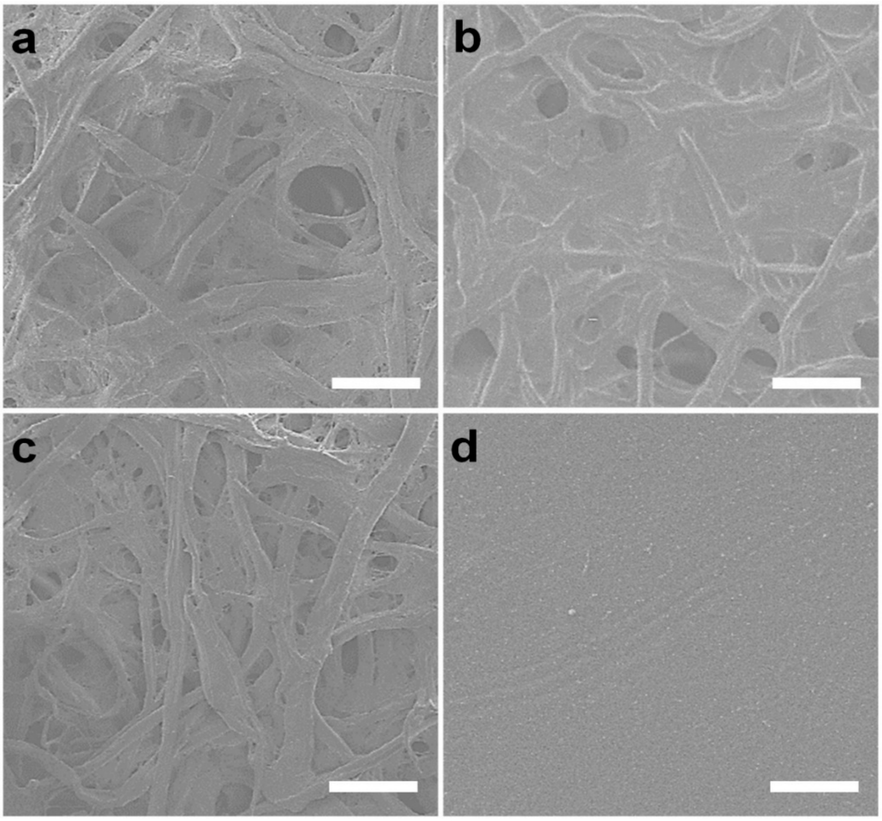

Figure S1. Surface image of cellulose fiber in paper showing changes caused by the photocurable resin during 3D digital light printing (DLP) and ethanol washing. (a) The bare filter paper. (b) The filter paper soaked with resin. (c) 10 times of ethanol washing after the filter paper was soaked with resin (d) The fully cured paper. (Scale bar: 50 µm. magnification: 100X). Images were obtained with Scanning electron microscopy (JSM-6700F, JEOL, Tokyo, Japan).

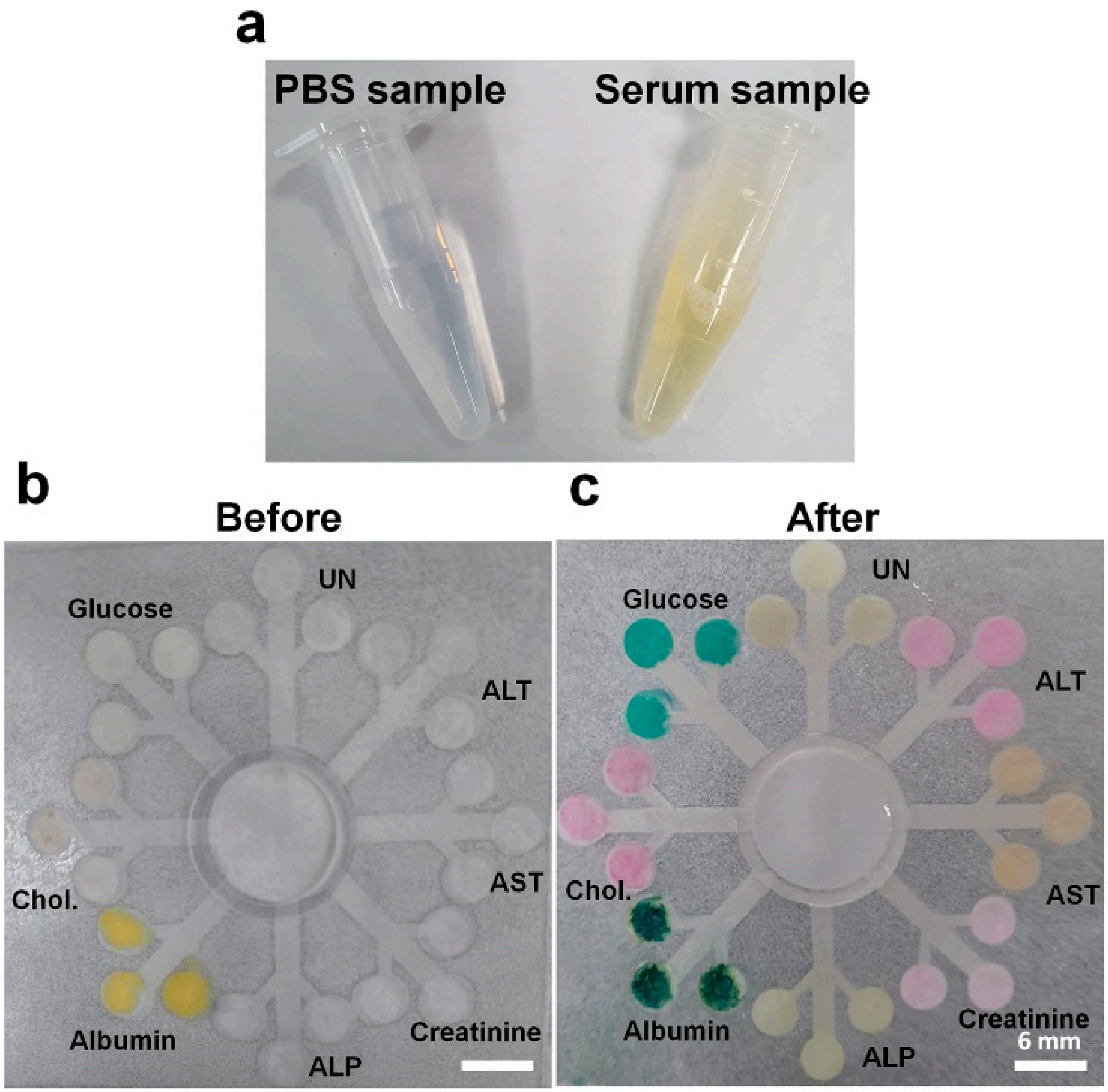

Figure S2. Multiple detection on the 3D-μPADs using a serum sample. (a) Color difference between the PBS sample and spiked serum sample in tubes. (b) Each enzyme was fixed on each detection zone before testing. (c). Detection of multiple biomarkers by dropping the sample (300 μL) including 8 mmol/L for glucose, 8 mmol/L for Chol., 4 g/dL for albumin, 400 U/L for ALP, 150 μmol/L for creatinine, 400 U/L for AST, 400 U/L for ALT, 4 mmol/L for UN in spike serum sample.

[1] C. Park, H.-R. Kim, S.-K. Kim, I.-K. Jeong, J.-C. Pyun, S. Park, Three-Dimensional Paper-Based Microfluidic Analytical Devices Integrated with a Plasma Separation Membrane for the Detection of Biomarkers in Whole Blood, ACS App. Mater. Interfaces 11(2019) 36428-36434. https://doi.org/10.1021/acsami.9b13644
